# Therapeutics

**Published:** 1876-07

**Authors:** 


### IV. THERAPEUTICS.

1. *Spider Web or Tela Araneæ in Chronic Intermittents.* L. M. JONES, M.D. (*Cin. Lancet and Observer*, May, 1876.)

Being at his wit's end, in treating a rebellious case of intermittent in a girl ten years old, with *quinine*, *camphor chinoidine pills*, *arsenic*, *eucalyptus*, *iodine*, phosphoric acid, etc., etc., with unsatisfactory results, Dr. Jones determined, upon Dr. Robert Jackson's recommendation in the U. S. Dispensatory, to use spider web. He then says :

"Accordingly I went to the drug store and had some prepared in the following manner : Went with the druggist to the cellar (as it is the species of spider that inhabits cellars or dark places, that possesses medicinal properties) and with a stick I gathered cobwebs until I had a wad or bunch the size of a large-hulled walnut.

"This we put in a bottle with four ounces of good whisky, which was allowed to macerate for forty-eight hours, when it was filtered, and the liquid poured into a bottle. I carried the medicine to my patient, and left the following directions: Begin four hours before the expected chill, and give a teaspoonful every hour, until she had taken four doses, then a teapoonful before each meal and at bedtime, until all was taken. The medicine was given as directed. The anticipated chill came, but was very light compared with the others. This, however, was the last chill that she had, which has been over four months since.

"Her general appearance has improved, color of skin is clear, the spleen has returned to nearly its natural size, bowels regular, appetite good ; in fact, the child has so far improved that her friends call her well.

"The patient and relatives did not know what she was taking, and are still ignorant of the subject, so that the mind of the patient had nothing to do in the performance of the cure.

"I gave her the second bottle of the medicine, to be certain that the cure was effectual.

"I have since used the remedy in other cases, with the same success, and would ask that the profession give the remedy a trial in the various intermittents. I will offer no comments on the case that I have cited, or give any theory as to the action of the remedy, but shall leave the reader to his own views. I give a history of this case because I considered it an interesting one in several particulars ; also was particular in giving the treatment, that a comparison might be made in the remedies used and the results obtained ; and that the reader might see for himself that the treatment adopted by the writer was a varied one. I will add, in conclusion, that either of the prescriptions given will break up an ordinary case of intermittent fever, chronic or not."

2. *Pomegranate Bark for Tape-worm.* PROF. EDWIN FREEMAN, M.D. (*Ec. Med. Jour.*, May, 1876.)

I succeeded in obtaining a tape worm entire, with its head, from W. S., of Avondale, which had resisted the use of a good many medicines during five years before I began to medicate its possessor for it. The worm obtained was thirty-four feet long, when it came away. Mr. S. said that he passed, at some past time, forty-two feet in one piece, but the head remained, and he has every day passed several inches, for at least three years. While he had the worm he was a large eater, and seemed to require, besides, a quart or two of coffee every morning. It caused him, at times, to be faint and dizzy, with occasional headache and weakness and sluggishness in the morning. In addition to this, there were occasionally nausea and fullness of the stomach. The best evidence of the presence of tape-worm is the passage, with the fæces, of portions of one.

*Treatment.*—I gave him, the first thing in the morning, two seidlitz powders, which thoroughly evacuated the bowels. I then gave him morphia sulph. gr. ⅛. In an hour he began to take the pomegranate, a decoction of the bark, four ounces every fifteen minutes, until it was all taken. The decoction was prepared by the chemist, J. U. Lloyd, from the best bark, according to the formula published by Prof. Locke, in a former number of the E. M. Journal, and mixed with fluid extract jalap, dr. j.

After the third dose the worm was felt to have lost his hold on the bowel, and to be low down. The fourth dose was not taken until an hour after the third, hoping that the worm would surrender and come away. It was stubborn, however, but at the last dose submitted unconditionally, like Davy Crockett's 'coon, coiled himself into a knot, and got down and out.

The dose is a fearful one to swallow, but for those who can take it, it is effectual in ridding them of a very annoying trouble.

3. *Choleate of Soda as a Preventive of Gall Stones.* WM. C. DABNEY, M.D. (*Amer. Jour. Med. Sci.*, April, 1876.)

The article and its use, indicated above, were recommended by Prof. Schiff, of Florence, in 1874. Dr. Dabney has used it in three well-marked cases of biliary calculi diatheses, with extremely satisfactory results. The attention of physicians who have under their care any patients suffering regularly from gall-stone colic, is called to this agent. It is used in 5 to 7-grain doses, twice daily, and is a perfect solvent of the cholesterine and mucus which compose the "stones." It will prevent future attacks of colic, but will not relieve a fit of it when it is once precipitated.

